# The difference in prolonged continuous and intermittent Pringle maneuver during complex hepatectomy for hepatocellular carcinoma patients with chronic liver disease: A retrospective cohort study

**DOI:** 10.1002/cam4.4361

**Published:** 2021-10-18

**Authors:** Jianwei Liu, Wei Wang, Chunchao Shi, Chenqi Li, Feng Xue, Lei Hu, Yi Wang, Ruiliang Ge

**Affiliations:** ^1^ Department of Hepatic Surgery II Eastern Hepatobiliary Surgery Hospital Naval Medical University Shanghai China; ^2^ Department of Colorectal Surgery Changhai Hospital Naval Medical University Shanghai China; ^3^ Department of Nutrition Eastern Hepatobiliary Surgery Hospital Naval Medical University Shanghai China; ^4^ Department of Outpatient Eastern Hepatobiliary Surgery Hospital Naval Medical University Shanghai China

**Keywords:** bleeding, complications, continuous Pringle maneuver, intermittent Pringle maneuver, liver function

## Abstract

**Purpose:**

To explore the differences between prolonged continuous Pringle maneuver (CPM) and prolonged intermittent Pringle maneuver (IPM) in patients with hepatocellular carcinoma (HCC), who underwent complex hepatectomy.

**Methods:**

This retrospective cohort study performed between June 2014 and May 2016 included 142 patients who underwent complex hepatectomy for HCC and concomitant chronic liver disease but with good liver function. Patients were categorized into CPM (*n* = 69) and IPM groups (*n* = 73). The differences in these aspects were compared between the two groups which include operation time, intraoperative bleeding, perioperative transfusion, postoperative complications, liver function injury, postoperative overall survival (OS), and tumor recurrence.

**Results:**

The cumulative clamping time, operation time, intraoperative bleeding, and perioperative transfusion rates were 38.0, 132 min, 300 ml, and 17.4% in CPM and 40.0, 145 min, 400 ml, and 32.9% in IPM, respectively. There were significant intergroup differences in operation time (*p* = 0.018), intraoperative bleeding (*p *< 0.001), and perioperative transfusion rates (*p* = 0.034). Besides, the postoperative complications and postoperative liver function injury of the CPM group were better than those of IPM. There was no significant intergroup difference in OS (*p* = 0.908) and tumor recurrence (*p* = 0.671) between two groups.

**Conclusion:**

Compared with IPM, CPM with a cumulative clamping time between 30 and 50 min can shorten operation time, reduce intraoperative bleeding and perioperative transfusion, and reduce postoperative complications and postoperative liver function injury in patients who underwent complex hepatectomy for HCC and concomitant liver disease but with good liver function. There was no significant difference in OS and tumor recurrence between two groups.

## Graphical abstract

This is the first study to explore the difference in prolonged continuous Pringle maneuver (CPM) and prolonged intermittent Pringle maneuver (IPM) in HCC patients who underwent complex liver resection. The results showed that CPM whose cumulative clamping time between 30 and 50 min can shorten operation time, reduce intraoperative bleeding and proportion of transfusion, and reduce the postoperative complications and postoperative liver function injury, similar to overall survival and tumor recurrence compared with IPM. Therefore, CPM should be preferred when the estimated clamping time was between 30 and 50 min for HCC patients with chronic liver disease and well‐compensated liver function.

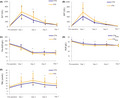

## 1 INTRODUCTION

Primary liver cancer is the seventh most common malignancy globally, and is associated with the highest morbidity and mortality among malignant tumors.^1^ The incidence of liver malignancies is high in China, and more than 50% of these cases occur in China every year.^2^ Hepatocellular carcinoma (HCC) is the most common primary liver malignancy and accounts for approximately 85% of all cases.^3^, ^4^ Radical hepatectomy is the most widely used treatment for HCC.^5^, ^6^ However, it is important to minimize intraoperative bleeding and the consequent need for transfusion, which remain the primary concerns associated with hepatectomy and serve as significant predictors of short‐ and long‐term outcomes.^7^, ^8^, ^9^

Hepatic blood inflow occlusion (the Pringle maneuver [PM]) reduces intraoperative bleeding and is therefore extensively used during hepatectomy. PM has been shown to be a safe and effective approach to control massive hemorrhage during hepatectomy^10^ and can be performed using continuous or intermittent clamping of the portal triad.^11^ However, ischemia‐reperfusion (IR) injury of the remnant liver parenchyma is a well‐known drawback of vascular clamping methods, particularly in patients with chronic liver disease.^12^, ^13^ The intermittent Pringle maneuver (IPM) involves short periods of reperfusion after continuous clamping of the hepatic portal and was shown to cause lesser IR injury compared with the continuous Pringle maneuver (CPM).^14^ However, IPM may cause additional bleeding during reperfusion.^15^ Many studies have investigated the effects of CPM or IPM within 30 min.^16^, ^17^ However, the differences between prolonged CPM and IPM remain unclear. Studies have reported that hepatic blood inflow occlusion may promote tumor recurrence and metastasis, thereby affecting the long‐term prognosis of patients with HCC.^18^ However, the effects of prolonged CPM or IPM on long‐term prognosis remain unclear. Research has shown that in patients with HCC (even in those with chronic liver disease), CPM can safely be performed up to 50 min without an increased risk of postoperative complications and perioperative mortality.^19^ In this study, we compared the difference between CPM and IPM with cumulative clamping time between 30 and 50 min on operation time, intraoperative bleeding, perioperative transfusion, postoperative complication, liver function injury, overall survival (OS), and tumor recurrence rates in patients with HCC and concomitant chronic liver disease but with good liver function.

## 2 METHODS

### 2.1 Study population and design

The study included patients who underwent complex hepatectomy at our hospital between June 2014 and May 2016. Complex liver resection was defined as an operation performed for tumors of large diameter that necessitate extensive hepatectomy or for tumors in the vicinity of large vessels, which are surgically challenging or require vascular reconstruction.^20^, ^21^ Inclusion criteria of this study were as follows: (i) HCC was confirmed by postoperative pathology; (ii) Diagnosis of chronic hepatitis or cirrhosis; (iii) Well‐compensated liver function, Child–Pugh grade A; (iv) Technically resectable and underwent complex liver resection; (v) Cumulative clamping time between 30 and 50 min; and (vi) No blood system disease, and/or abnormalities of coagulation function. Exclusion criteria were as follows: (i) Outflow occlusion, including vena cava clamping and hepatic vein occlusion and (ii) Concomitant major surgical procedures or procedures such as bilioenteric anastomosis or associated gastrointestinal procedures and intraoperative radiofrequency ablation, which tend to affect the operation time, intraoperative bleeding, perioperative transfusion, postoperative complications, postoperative liver function injury, OS, or tumor recurrence rates. The Ethics Committee of our hospital approved this study. All patients obtained informed consent before surgery.

### 2.2 Preoperative auxiliary examination and hepatectomy

Routine examination was performed before operation, which include laboratory tests and imaging examinations. Laboratory tests include routine blood tests, and tests for liver, kidney and coagulation function, tumor markers, viral hepatitis, HBV‐DNA level, routine urine and stool analysis, tests for syphilis and HIV virus, as well as blood group screening. Imaging examinations included abdominal ultrasonography, liver magnetic resonance imaging (MRI), and multislice computed tomography (CT). Patients’ general health condition, liver function, and the technical feasibility of the surgery were considered before planning hepatectomy. Chronic liver disease was evaluated based on laboratory tests and imaging examinations, including serological testing for viral hepatitis and liver function.

Following induction of general anesthesia with endotracheal intubation, an oblique or reverse L‐shaped incision was created under the right costal margin. After the abdomen was opened, we performed intraoperative ultrasonography to evaluate the liver anatomy and confirm the extent of the intrahepatic tumor and any major vascular invasion. Subsequently, the ligaments on the liver surface were detached at their attachments, and the liver was completely exposed. The PM was performed following complete exposure of the first porta hepatis, and using an electrosurgical knife, we marked a parenchymal transection line after which we used an ultrasonic scalpel and clamp crushing technique for liver parenchymal transection. The hepatic artery, portal vein, and bile duct were ligated using silk sutures and were transected during liver parenchyma disconnection. Anatomical or non‐anatomical hepatectomy was performed to ensure a negative surgical margin. In some patients, the cut surface of the liver was sutured using different types of silk sutures after complete resection of the liver tumor; we avoided excessive tension during suture application to prevent injury to the liver tissue.

The PM is classified into the CPM and IPM types. IPM refers to intermittent clamping of the portal triad and involves multiple cycles of 15‐min clamp time and 5‐min release of the portal triad. CPM refers to continuously clamping the portal triad. In case the clamping time was exceeding 50 min, the occlusion was released 5 min. During this process, surgical gauze was used to cover the cut surface of the liver to reduce the volume of bleeding. Re‐occlusion was performed after a 5‐min release time. These patients were not included in this study according to the inclusion criteria.

### 2.3 Postoperative management and follow‐up

All patients received similar postoperative care and treatment in the general surgical ward. Parameters of liver function, including serum alanine aminotransferase (ALT), aspartate aminotransferase (AST), total bilirubin (TBIL), albumin (ALB), and pre‐albumin (pre‐ALB) levels, were measured on postoperative days 1, 3, 5, and 7. Postoperative complications were graded based on the Clavien–Dindo classification.^22^ Liver dysfunction was defined using the “50–50 criteria”.^23^

All patients underwent regular 3‐month post‐hepatectomy follow‐up during the first 2 years and 3–6 months of follow‐up thereafter. Serum alpha fetoprotein (AFP), carcinoembryonic antigen, carbohydrate antigen 19–9, liver function, renal function evaluation, as well as abdominal ultrasonography/or MRI were performed at each follow‐up visit. OS was from the date of operation to the date of last follow‐up or death, and recurrence time was from the date of operation to the date of the first diagnosis of recurrence or metastasis.

### 2.4 Statistical analysis

Data were analyzed using the IBM SPSS Statistics for Windows software, version 26.0 (IBM Corp.). Continuous variables were expressed as median (IQR, interquartile range) and intergroup differences were compared using the *t*‐test or the Mann–Whitney *U* test. Categorical variables were expressed as frequencies (percentages) and the intergroup differences were compared using the chi‐squared test or the Fisher's exact test. The Kaplan–Meier method was used to draw the survival curves of OS and tumor recurrence. The independent risk factors of OS and tumor recurrence were analyzed by Cox multivariate analysis. *P* < 0.05 was considered statistically different.

## 3 RESULTS

### 3.1 Patient characteristics

**FIGURE 1:**
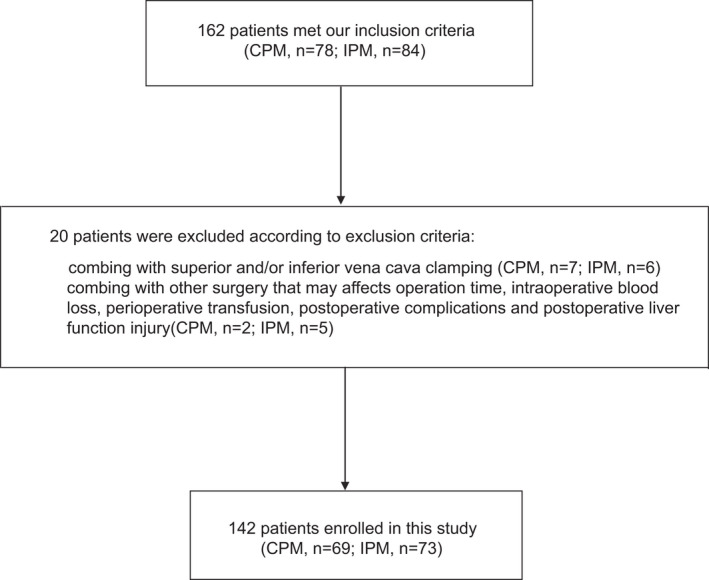
The flow chart of this study

Figure 1 shows the flow chart of this study, which initially included 162 patients. Thirteen patients were excluded because of superior and/or inferior vena cava clamping intraoperatively (seven in CPM group and six in IPM group). Seven patients were excluded who underwent combined surgery that could may affect operation time, intraoperative bleeding, perioperative transfusion, postoperative complications, and postoperative liver function injury (two in CPM group and five in IPM group). Therefore, finally, 142 patients who underwent complex hepatectomy for HCC and concomitant chronic liver disease but with good liver function were enrolled in this study. Among them, 69 and 73 patients underwent CPM and IPM, respectively.

Table 1 shows no intergroup differences in age, sex, TBIL, ALT, AST, Alb, Pre‐Alb, prothrombin time, platelet count, AFP, HBV‐DNA level, etiology of the liver disease, major hepatectomy, anatomic hepatectomy, chronic hepatitis and cirrhosis, tumor number and size, tumor capsule, microvascular invasion (MVI), and the Edmondson–Steiner grade (*p* > 0.05). The median cumulative clamping times were 38 and 40 min in the CPM and IPM groups, respectively, with no significant difference (*p* = 0.112). There was no significant intergroup difference in the post‐treatment hospital stay (*p* = 0.145). The operation time, intraoperative bleeding, and perioperative transfusion rates in CPM group were 132 min, 300 ml, and 17.4%, respectively, which were 145 min, 400 ml, and 32.9% in IPM group. There was significant difference between CPM and IPM (*p* = 0.018, *p* < 0.001, *p* = 0.034). Forty‐two (57.5%) patients in the IPM group and 23 patients (33.3%) in the CPM group had a close hepatic cutting surface during manipulation of the hepatic remnant facet, and this intergroup difference was statistically significant (*p* = 0.004).

**TABLE 1:** Clinical characteristics of patients who underwent hepatectomy with CPM or IPM

Variable	Number (%)/median (IQR)		*p*
	CPM (*n* = 69)	IPM (*n* = 73)	
Demographics			
Age, years	50.0 (42.5–59.0)	52.0 (44.5–61.0)	0.567
Sex, male	64 (92.8)	69 (94.5)	0.930
Preoperative laboratory tests			
TBIL, μmol/L	12.0 (10.1–15.4)	13.1 (9.6–18.0)	0.464
ALT, U/L	35.0 (26.6–53.0)	38.0 (25.3–67.0)	0.298
AST, U/L	37.0 (26.1–46.1)	37.0 (27.4–50.5)	0.431
ALB, g/L	42.0 (39.6–45.6)	41.4 (39.3–43.5)	0.189
Pre‐ALB, mg/L	210.0 (179.5–267.0)	208.0 (176.5–244.5)	0.351
PT, seconds	11.8 (11.3–12.4)	11.9 (11.4–12.8)	0.314
PLT, 10^9^/L	177.0 (117.5–230.0)	155.0 (114.5–195.0)	0.140
AFP, μg/L	103.0 (7.1–4523.5)	31.2 (5.9–943.1)	0.148
HBV‐DNA level, IU/ml, ≥2000	20 (29.0)	32 (43.8)	0.066
Etiology of liver disease			0.710
HBV	66 (95.7)	70 (95.9)	
HCV	1 (1.4)	2 (2.7)	
HBV+HCV	2 (2.9)	1 (1.4)	
Operative characteristics			
Cumulative clamping time, minutes	38.0 (34.0–43.0)	40.0 (35.0–45.0)	0.112
Operation time, minutes	132.0 (96.0–178.5)	145.0 (116.5–193.0)	0.018
Major hepatectomy^a^, yes	38 (55.1%)	44 (60.3%)	0.531
Hepatectomy, anatomic	35 (53.8%)	30 (46.2%)	0.250
Intraoperative bleeding, ml	300 (200–400)	400 (300–600)	<0.001
Perioperative transfusion^b^, yes	12 (17.4)	24 (32.9)	0.034
Close hepatic cutting surface, yes	23 (33.3)	42 (57.5)	0.004
Pathology characteristics			
Histology of nontumorous liver			0.263
Chronic hepatitis	46 (66.7)	42 (57.5)	
Cirrhosis^c^	23 (33.3)	31 (42.5)	
Tumor size^c^, cm	8.5 (6.9–11.0)	9.0 (7.6–10.6)	0.347
Tumor number^c^, multiple	17 (24.6)	20 (27.4)	0.708
Tumor capsule^c^, incomplete	40 (58.0)	44 (60.3)	0.780
MVI^c^, presence	15 (21.7)	26 (35.6)	0.068
Edmondson–Steiner grade^c^, III/IV	60 (87.0)	62 (84.9)	0.729
Post‐treatment hospital stay, days	8.0 (8.0–9.0)	8.0 (9.0–10.0)	0.145

Abbreviations: AFP, alpha fetoprotein; ALB, albumin; ALT, alanine aminotransferase; AST, aspartate aminotransferase; CPM, continuous Pringle Maneuver; HBV, hepatitis B virus; HCV, hepatitis C virus; IPM, intermittent Pringle Maneuver; IQR, interquartile range; MVI, microvascular invasion; PLT, platelet count; Pre‐ALB, pre‐albumin; PT, prothrombin time; TBIL, total bilirubin.

^a^ Resection of ≥3 Couinaud’s hepatic segments.

^b^ Including red blood cell suspension and plasma infused during perioperative period.

^c^ Based on pathology.

### 3.2 Comparison of postoperative liver function between the CPM and IPM groups

**FIGURE 2:**
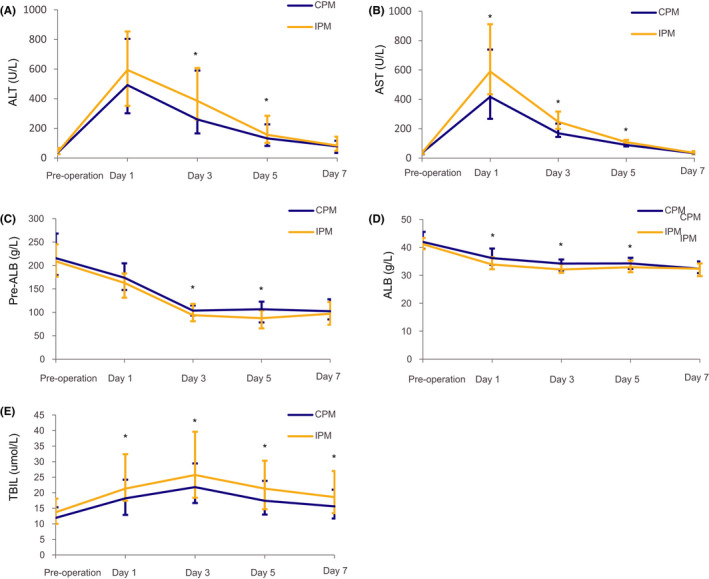
(A) The fluctuations of ALT on pre‐operation, postoperative days 1, 3, 5, and 7; (B) The fluctuations of AST on pre‐operation, postoperative days 1, 3, 5, and 7; (C) The fluctuations of Pre‐ALB on pre‐operation, postoperative days 1, 3, 5, and 7; (D) The fluctuations of ALB on pre‐operation, postoperative days 1, 3, 5, and 7; and (E) The fluctuations of TBIL on pre‐operation, postoperative days 1, 3, 5, and 7

Figure 2 depicts the fluctuations of liver function parameters (including ALT, AST, ALB, pre‐ALB, and TBIL) on postoperative days 1, 3, 5, and 7. The AST and TBIL levels were lower and ALB levels were higher (*p* < 0.05) in the CPM group than in the IPM group, along with similar ALT and pre‐ALB levels on postoperative day 1 (*p* > 0.05). The ALT, AST, and TBIL levels were lower and ALB and pre‐ALB levels were higher in the CPM group than in the IPM group on postoperative day 3 (*p* = 0.019, 0.001, 0.035, 0.002, 0.038) and day 5 (*p* = 0.035, 0.024, 0.031, 0.014, 0.002). No significant intergroup difference was observed in the liver function on postoperative day 7, except for serum TBIL level (*p* = 0.042; Table 2; Figure 2).

**TABLE 2:** Perioperative liver function parameters of patients who underwent hepatectomy with CPM or IPM

Variable	CPM (*n* = 69)	IPM (*n* = 73)	*p*
ALT, U/L			
Pre‐operation	35.1 (26.3–53.0)	38.0 (25.9–67.0)	0.298
Postoperative day 1	491.9 (302.1–806.8)	594.1 (352.0–854.0)	0.250
Postoperative day 3	259.5 (165.6–590.3)	385.5 (258.8–607.0)	0.019
Postoperative day 5	131.9 (81.7–227.0)	156.7 (101.8–285.3)	0.035
Postoperative day 7	78.7 (44.3–104.8)	83.8 (56.8–128.7)	0.124
AST, U/L			
Pre‐operation	36.5 (26.1–46.0)	37.0 (27.6–51.0)	0.431
Postoperative day 1	417.5 (267.1–739.3)	590.1 (435.0–912.1)	0.004
Postoperative day 3	169.0 (111.3–232.5)	247.0 (139.3–354.8)	0.001
Postoperative day 5	89.8 (70.0–106.5)	109.0 (72.0–135.4)	0.024
Postoperative day 7	33.9 (27.1–42.2)	36.0 (28.8–47.1)	0.153
ALB, g/L			
Pre‐operation	42.0 (39.5–45.6)	41.2 (39.3–43.4)	0.189
Postoperative day 1	36.2 (33.8–39.6)	33.9 (32.2–36.5)	<0.001
Postoperative day 3	34.2 (31.8–35.7)	32.1 (30.9–34.1)	0.002
Postoperative day 5	34.3 (32.3–36.3)	32.9 (31.1–35.3)	0.014
Postoperative day 7	32.4 (30.8–35.0)	32.4 (29.7–34.3)	0.403
Pre‐ALB, g/L			
Pre‐operation	215.5 (179.3–268.0)	209.0 (176.0–245.0)	0.351
Postoperative day 1	174.0 (147.8–204.5)	163.0 (131.0–183.0)	0.075
Postoperative day 3	103.5 (93.3–114.8)	94.0 (81.0–118.0)	0.038
Postoperative day 5	106.7 (78.3–122.6)	87.3 (65.7–104.4)	0.002
Postoperative day 7	102.2 (85.0–127.9)	96.8 (73.7–122.1)	0.075
TBIL, μmol/L			
Pre‐operation	12.0 (10.1–15.4)	13.1 (9.6–18.0)	0.464
Postoperative day 1	18.6 (13.0–34.9)	21.2 (16.9–30.7)	0.013
Postoperative day 3	21.4 (16.7–29.4)	25.7 (18.4–39.0)	0.035
Postoperative day 5	17.3 (13.0–23.7)	21.3 (14.4–30.7)	0.031
Postoperative day 7	15.6 (11.7–21.0)	18.6 (13.4–27.0)	0.042

Abbreviations: ALB, albumin; ALT, alanine aminotransferase; AST, aspartate aminotransferase; Pre‐ALB, pre‐albumin; TBIL, total bilirubin.

### 3.3 Comparison of postoperative complications between the CPM and IPM groups

Table 3 shows a comparison of the postoperative complications, including hepatic insufficiency, pleural effusion, ascites, fever (>38.5°C, >3 days), intra‐abdominal hemorrhage and infection, bile leakage, pneumonia, wound infection, gastrointestinal hemorrhage, atelectasis, or pneumothorax. In the CPM group, 17 patients had complications: 11 patients had one complication, five patients had two different complications, and one patient had three different complications. Of the 17 patients, two patients had Grade III/IV complications. In the IPM group, 31 patients had complications: 23 patients and eight patients had one and two different complications, respectively. Of the 31 patients, three patients had Grade III/IV complications. The incidence of postoperative complications in the CPM and IPM groups was 24.6% and 42.5%, respectively, and the incidence of Grade III/IV complications was 2.9% and 4.1%, correspondingly. The overall complication rate was lower in the CPM group than that in the IPM group (*p* = 0.037). However, the rate of Grade III/IV complications had no significant intergroup difference (*p* = 0.694). There was also no perioperative death in the two groups.

**TABLE 3:** Complication of patients who underwent hepatectomy with CPM or IPM

Complication	All grade (*n*)^b^			Grade III/IV (*n*)^b^		
	CPM (*n* = 69)^a^	IPM (*n* = 73)^a^	*p*	CPM (*n* = 69)^a^	IPM (*n* = 73)^a^	*p*
Overall complication	17^c^	31^e^	0.037	2^d^	3^f^	0.694
Hepatic insufficiency^g^	4	8		0	0	
Pleural effusion	3	5		1	1	
Ascites	4	7		0	0	
Fever (>38.5°C, >3 days)	3	4		0	0	
Intra‐abdominal hemorrhage	2	3		0	0	
Intra‐abdominal infection	2	4		0	0	
Bile leakage	2	3		0	1	
Pneumonia	1	1		0	0	
Wound infection	1	1		0	0	
Gastrointestinal hemorrhage	2	2		1	1	
Atelectasis or pneumothorax	0	1		0	0	

^a^ Patients who actually received CPM or IPM were analyzed.

^b^ According to the Clavien–Dindo classification.

^c^ Eleven patients had one complication, five patients had two different complications, and one patient had three different complications.

^d^ Two patients had one complication.

^e^ Twenty‐three patients had one complication and eight patients had two different complications.

^f^ Three patients had one complication.

^g^ Liver dysfunction was defined using the “50–50 criteria”.

### 3.4 Comparison of postoperative OS and tumor recurrence between the CPM and IPM groups

The median follow‐up time in this study was 61.8 months and the range was from 7.3 to 70.4 months. The postoperative 1‐, 3‐, and 5‐year OS rates were 86.6%, 59.2%, and 41.5%, respectively, and the postoperative 1‐, 3‐, and 5‐year tumor recurrence rates were 25.4%, 63.4%, and 79.6%, respectively.

**FIGURE 3:**
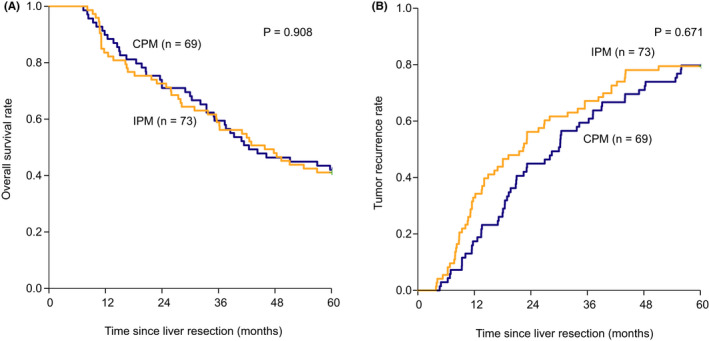
(A) Kaplan–Meier curves of OS for HCC patients who underwent CPM or IPM during complex hepatectomy and (B) Kaplan–Meier curves of tumor recurrence for HCC patients who underwent CPM or IPM during complex hepatectomy

Table 4 shows the results of univariate Cox regression analysis. Table 5 shows the results of multivariate Cox regression analysis. Multivariate analysis showed that AFP ≥20 µg/L (hazard ratio [HR] 2.307, 95% confidence interval [CI] 1.445–3.682), multiple tumor (HR 2.471, 95%CI 1.555–3.928), and MVI (HR 3.024, 95%CI 1.856–4.925) were independent risk factors for OS and AFP ≥20 µg/L (HR 1.789, 95%CI 1.208–2.649), multiple tumors (HR 1.739, 95%CI 1.148–2.633), MVI (HR 1.900, 95%CI 1.239–2.915) were independent risk factors for tumor recurrence. The results showed that CPM was not an independent risk factor for OS or tumor recurrence. The postoperative 1‐, 3‐, and 5‐year OS rates were 89.9%, 59.4%, and 42.0% and 83.6%, 58.9%, and 41.1% for CPM and IPM (*p* = 0.908), respectively. And the postoperative 1‐, 3‐, and 5‐year tumor recurrence rates were 17.4%, 59.4%, and 79.7% and 32.9%, 67.1%, and 79.5% for CPM and IPM (*p* = 0.671), respectively. (Figure 3).

**TABLE 4:** Univariate analysis of OS and tumor recurrence

Variable	OS			Tumor recurrence		
	*p*	HR	95%CI	*p*	HR	95%CI
Age, years, ≥60	0.077	0.595	0.335–1.058	0.599	0.886	0.564–1.391
Sex, male	0.627	0.825	0.380–1.792	0.611	1.220	0.567–2.623
TBIL, µmol/L, ≥17.1	0.642	1.124	0.686–1.843	0.377	1.210	0.793–1.845
ALT, U/L, >40	0.148	1.375	0.894–2.115	0.057	1.437	0.989–2.087
AST, U/L, ≥40	0.089	1.451	0.945–2.229	0.097	1.369	0.945–1.983
ALB, g/L, ≥35	0.697	0.914	0.580–1.439	0.111	0.729	0.494–1.075
Pre‐ALB, mg/L, ≥280	0.717	0.902	0.516–1.577	0.990	1.003	0.624–1.613
PT, S, ≥13	0.350	0.808	0.517–1.263	0.893	0.974	0.667–1.422
PLT, 10^9/L, ≥100	0.955	1.018	0.552–1.875	0.833	0.945	0.557–1.603
AFP, µg/L, ≥20	0.008	1.848	1.172–2.912	0.021	1.575	1.072–2.315
HBV DNA, IU/ml, ≥2000	0.605	0.888	0.566–1.393	0.545	0.888	0.603–1.306
Pringle maneuver, CPM	0.908	0.974	0.623–1.522	0.671	1.087	0.740–1.595
Hepatectomy, major	0.443	1.187	0.766–1.839	0.995	0.999	0.688–1.451
Hepatectomy, non‐anatomic	0.387	0.826	0.536–1.273	0.160	0.765	0.527–1.111
Blood transfusion	0.191	1.372	0.854–2.205	0.394	1.202	0.788–0.833
Cirrhosis	0.968	1.009	0.650–1.568	0.624	0.909	0.620–1.332
Tumor diameter, cm, ≥5	0.824	1.172	0.288–4.768	0.383	1.865	0.460–7.560
Tumor number, multiple	<0.001	2.350	1.499–3.686	0.022	1.607	1.070–2.414
Tumor capsule, incomplete	0.022	1.703	1.081–2.682	0.041	1.492	1.017–2.191
MVI, presence	<0.001	2.697	1.724–4.220	0.003	1.837	1.225–2.753
Edmondson–Steiner grade, III/IV	0.519	0.823	0.456–1.487	0.799	1.073	0.623–1.849
Complication	0.497	0.852	0.536–1.353	0.359	0.829	0.555–1.237

Abbreviations: AFP, alpha fetoprotein; ALB, albumin; ALT, alanine aminotransferase; AST, aspartate aminotransferase; CPM, continuous Pringle Maneuver; HBV‐DNA, hepatitis B virus DNA; MVI, microvascular invasion; PLT, platelet count; Pre‐ALB, pre‐albumin; PT, prothrombin time; TBIL, total bilirubin.

**TABLE 5:** Multivariate analysis of OS and tumor recurrence

Variable	OS			Tumor recurrence		
	*p*	HR	95%CI	*p*	HR	95%CI
AFP, µg/L, >20	<0.001	2.307	1.445–3.682	0.004	1.789	1.208–2.649
Tumor number, multiple	<0.001	2.471	1.555–3.928	0.009	1.739	1.148–2.633
Tumor capsule, incomplete	0.230	3.024	1.856–4.925	0.133	1.359	0.911–2.026
MVI, presence	<0.001	3.024	1.856–4.925	0.003	1.900	1.239–2.915

Abbreviations: AFP, alpha fetoprotein; MVI, microvascular invasion.

## 4 DISCUSSION

Minimizing and controlling intraoperative bleeding has always been the most important issue throughout the history of liver surgery. Massive intraoperative bleeding and transfusion can precipitate postoperative complications and are significantly associated with perioperative mortality.^7^, ^8^ The PM is an effective and commonly used strategy to control intraoperative hemorrhage.^24^ In Japan, 25% of surgeons use the PM during hepatectomy, even in patients with cirrhosis^25^; reportedly, >70% of European hepatic surgeons perform vascular clamping.^26^ Unfortunately, these clamping methods can result in ischemia‐reperfusion (IR) injury to the remnant liver. An earlier study reported that regenerative nodules in the cirrhotic liver are more reliant on arterial blood supply, which may increase their sensitivity to hypoxia.^27^ In other words, the risk of IR injury is higher in the cirrhotic liver than in a normal liver.^28^ Prolonging the clamping time to control intraoperative bleeding during hepatectomy and simultaneously limiting IR injury to the remnant liver parenchyma presents a surgical dilemma. Achieving the balance between intraoperative bleeding and IR injury has always been the focus of clinical research. The currently prevalent viewpoint is that the liver can tolerate 30 or even 50 min of clamping‐induced normothermic ischemia even in patients with cirrhosis.^16^, ^19^, ^29^

The PM can be classified into CPM and IPM. IPM, first reported in 1987 by Makuuchi et al.,^30^ is a proven strategy to prevent prolonged ischemia.^12^, ^28^ However, the IPM may increase the risk of bleeding from the transected surface of the liver during each reperfusion period^12^ and also prolongs the operation time. Currently, the effects of CPM and IPM during hepatectomy on the intra‐ and postoperative conditions of hepatectomy remain controversial.^10^, ^13^, ^31^ Most studies have investigated the effects of CPM or IPM during hepatectomy with clamping times within 30 min.^16^, ^17^ However, complex hepatectomy performed for HCC accompanied by chronic liver disease is a relatively longer procedure with a corresponding increase in the cumulative clamping and operation times. Whether intra‐ and perioperative outcomes and long‐term prognosis differ between patients with chronic liver disease but with good liver function who undergo prolonged CPM and prolonged IPM (30–50 min) remains unclear, and currently, there is lack of research on this issue. In this study, we systematically compared the differences between prolonged CPM and IPM with regard to the cumulative clamping and operation times, intraoperative bleeding, and the perioperative transfusion, postoperative complication, postoperative liver injury, OS, and tumor recurrence rates. In our study, included patients with HCC and chronic liver disease, including chronic hepatitis or cirrhosis, and well‐compensated liver function. We found that compared with IPM, CPM with ischemia time of 30–50 min was safe and did not increase the risk of postoperative liver injury. Contrary to our expectation, the CPM group had a better liver function within a short time postoperatively compared with IPM group, which may be attributable to lesser intraoperative bleeding and a lower perioperative transfusion rate in CPM group than in IPM group (*p *< 0.001, *p* = 0.034). The CPM group showed lesser intraoperative bleeding, and hemostasis was easier during the clamping phase because of better visualization of the surgical field. However, the hemostasis in the IPM group was usually achieved during the reperfusion period, which is associated with serious blood oozing and therefore poor visualization of the operative field. It was easier to ignore the minor bleeding point. The hepatic cutting surface was more likely to be closed hastily for achieving rapid hemostasis, which may increase the risk of postoperative bleeding and the consequent need for transfusion. Excessive bleeding and consequent transfusion can lead to hemodynamic instability and liver function injury.^8^, ^10^, ^29^ Our study also showed that the percentage of close hepatic cutting surface was significantly higher in IPM group than in CPM group (*p* = 0.004), which indicates that the venous return in the remnant liver may be affected at the time the hepatic cutting surface was closed and is likely to affect postoperative liver function, indicated by serum TBIL, ALT, AST, ALB, and pre‐ALB levels. The results of this study also showed that the operative time of CPM group was shorter than that in IPM group, which may lead to shorter anesthesia time and lower postoperative infection rate.^32^ In our study, there was no difference in perioperative mortality between CPM and IPM groups, which may be attributable to the advances in surgical technology, particularly in the approaches to hepatectomy and strict preoperative patient screening.^33^, ^34^ However, the rate of postoperative complications was higher in the IPM group than in the CPM group, which could be attributable to the greater volume of intraoperative bleeding and higher rate of transfusion. Previous studies reported that higher incidence of complications was due to higher blood loss and transfusions.^7^, ^8^ The higher postoperative complication rate in the IPM group may also be explained by the longer operation and anesthesia times in these patients. With regard to prognosis, AFP ≥20 µg/L, multiple tumor, and MVI were independent risk factors for OS and tumor recurrence. There was no significant intergroup difference in OS and tumor recurrence between the CPM and IPM group. In other word, compared with the effects of IPM, CPM had no negative effect on survival. Furthermore, tumor diameter also had no negative effect on survival in this study. This may be associated with the patients included in this study. All patients included in this study had large tumor diameter (tumor diameter ≥5 cm) except for four patients (tumor diameter <5 cm). A small number of patients with tumor diameter <5 cm make the data not comparable, which may be the reason that tumor diameter is not an independent risk factor for prognosis.

The limitation of this study was that it was a single‐center study. Besides, the sample size of this study was small even if the statistical results under this sample size are clear, which need large‐scale prospective studies to further confirm.

## 5 CONCLUSION

Compared with IPM, CPM whose cumulative clamping time between 30 and 50 min can shorten operation time, reduce intraoperative bleeding and transfusion, and reduce the postoperative complications and postoperative liver function injury in patients treated with complex hepatectomy for HCC and concomitant chronic liver disease but with good liver function. Besides, CPM has no significant effect on the prognosis compared with IPM. Therefore, CPM should be recommended when the estimated clamping time is between 30 and 50 min for patients who undergo hepatectomy for HCC and concomitant chronic liver disease but with good liver function.

## CONFLICT OF INTEREST

None.

## Data Availability

The data that support the findings of this study are available upon request from the corresponding author. The data are not publicly available due to privacy or ethical restrictions.
